# Developing Residents as Medical Educators via the McMaster Multidisciplinary Academic Day Planning Committee

**DOI:** 10.7759/cureus.5855

**Published:** 2019-10-07

**Authors:** Alexander G Chorley, J Mark Walton, Brenda Montesanto, Teresa M Chan

**Affiliations:** 1 Emergency Medicine, McMaster University, Hamilton, CAN; 2 Pediatric Surgery, McMaster University, Hamilton, CAN; 3 Postgraduate Medical Education, McMaster University, Hamilton, CAN

**Keywords:** leadership development, postgraduate medical education, multi-disciplinary

## Abstract

Background

Residents are being asked to perform educator roles such as curriculum design and learner assessment with minimal professional development in leadership or medical education. The Multidisciplinary Academic Day (MAD) planning committee is a resident-led initiative responsible for delivering combined educational half-day sessions and workshops for all residents at McMaster University.

Objective

We sought to determine the impact participation in MAD planning committee had on residents' professional development and career goals.

Methods

We conducted a program evaluation survey of 19 of 30 (63.3%) current and former committee members to determine how the MAD planning committee’s alumni perceived its usefulness, and how participation affected their professional development.

Results

Residents cited a desire to gain medical education experience, learn about event planning and management, and improve resident education as reasons for joining the committee; 89.5% of respondents felt they had met these goals. Experience on the committee included skills related to curriculum design, developing needs assessments and programmatic evaluation. Many residents felt it helped them acquire leadership skills such as decision-making, idea generation, delegation, and public speaking. Several noted that it had sparked an interest in medical education as part of an academic career, and one former member subsequently pursued a Master’s of Education. The majority of the respondents (78.9%) felt it was helpful for their careers and 94.7% would recommend this experience to others interested in leadership and medical education.

Conclusion

Involvement in the MAD planning committee is a highly useful way for residents to acquire leadership skills, develop an interest in medical education and work in a multidisciplinary team.

## Introduction

Residents are essential teachers of both medical students and other residents throughout medical training programs [1]. Residents are being asked to be leaders and educators, going beyond merely teaching. Senior residents often list the following educator roles as part of their adjunctive duties: design and implementation of curricula, assessment and remediation of junior residents, and assuming leadership roles within residency programs [2]. Similarly, junior faculty members have identified the areas of educational research, lecture development, interpersonal and leadership skills as areas where they require more professional development as new attending physicians [3]. Faculty who do participate in professional development take on more educational leadership roles, as a result, implement educational innovations and place an increased emphasis on educational scholarship. Longitudinal interventions, in particular, seem to promote the formation of networks and collaborations among participants. Participants also reported acquiring new skills related to leadership, teamwork and organizational skills [4].

Recognizing that the role of the educator begins before starting as faculty, we believe there is a role for "pre-faculty development" to give residents exposure and experience in the realm of medical education. To fill this gap in this professional development and spark interest in a career involving medical education, here we describe an educational program at McMaster University to develop residents as medical educators.

## Materials and methods

Our innovation

The Multidisciplinary Academic Day (MAD) planning committee was formed in 1997 by the Post-Graduate Medical Education (PGME) office to deliver combined educational sessions for residents in all specialties at McMaster University. The committee was originally led by a faculty member with minimal, token participation by trainees (e.g. trainees were used as a focus group to review pre-selected choices of speakers). Over the past five years, it has transitioned to a resident-led committee with a faculty advisor. The MAD team develops and delivers several half-day sessions and other workshops over the course of the academic year. The goal of these sessions is to deliver content related to the dominant training paradigm in Canada (the CanMEDS roles, with a focus on the intrinsic or “non-medical expert” roles) that is applicable to all specialties [5].

Committee Functioning

The committee is led by a resident who has typically had at least one year of experience with MAD and is democratically elected by the rest of the committee for a one-year term. New members are recruited at the beginning of each academic year by an open call via institutional email to all specialties. Candidates are screened to ensure their interests align with the committee's purpose, but essentially all who show interest are invited to the group.

The group meets in September to brainstorm ideas for the year and divides into subcommittees (of two to five people) responsible for each of the following 1) needs assessments, 2) programming and speaker recruitment, 3) developing and executing a publicity plan (including posters, emails, social media, trailers), 4) organizing logistics and 5) program evaluation. The group essentially divides out the tasks as described by Kern and colleagues in their classic text on curriculum design [6]. Less experienced residents are selected to head each subcommittee and report to the chair. The educational sessions for the year are outlined and the residents recruit speakers. One or two residents also act as Master of Ceremonies for the event, which hosts approximately 300 trainees (see Table 1).

**Table 1:** MAD events MAD - Multidisciplinary Academic Day

Academic Year	Dates	Topic
2018/2019	December 2018	The Human Physician
	September 2018	Mini-MAD - Legal Matters & Lawsuits
	May 2019	Mini-MAD - Physicians as Value Added in a Multidisciplinary Environment
2017/2018	December 2017	The Future of Medicine
	September 2017	Mini-MAD - Leadership
	April 2018	Mini-MAD - How Working in Healthcare Improves and Impairs Self-care
2016/2017	December 2016	Find Your Spark - Inspiration in Medicine
	March 2017	Mini-MAD - How residents are making a global impact on health
2015/2016	December 2015	Stop the Stigma: Inspiring Better Health Care in our Communities
	March 2016	Patient Safety
2014/2015	December 2014	Resilience in Medicine
	April 2015	MEDeD 101: Future of MedEd, How to Give a Presentation, Teaching in the Operating Room, Technology & Education, Learning How to Learn, Giving & Receiving Feedback
2013/2014	December 2013	Sometimes Fate can be a Game Changer
	April 2014	Organ Donation
2012/2013	October 2012	MAD Scholar Research Day
	December 2012	Resident Fatigue: Managing Healthcare Resources
	February 2013	Advocacy
	May 2013	Transition to Practice
2011/2012	October 2011	Medico-legal
	December 2011	It’s All About You: Physician Well-being
	February 2012	Opioids, Where They go Wrong & Where They go Right
	May 2012	Transition to Practice
2010/2011	October 2010	Creative Solutions: Meeting the Needs of the Under-serviced Populations
	December 2010	Physician Well-being
	February 2011	If I Could Teach Other Services One Thing
	May 2011	Disaster Management

Theory Behind the Committee Design

The MAD planning committee structure is built upon the principles described by Lave and Wenger around situated learning [7]. The faculty advisor, a seasoned Clinician Educator with graduate-level training, acts as a mentor and resource for the committee chair, giving advice and feedback on leadership style, running meetings and managing a team. The chair, in turn, advises the more junior residents on how to accomplish their goals and manage their subcommittees. In this way, the MAD planning committee functions as a community of practice (CoP) as outlined by Lave and Wenger [7].

The residents on this committee have created a self-selected community with a common goal of creating high-quality educational content for all residents at the university. As a multidisciplinary group from a variety of backgrounds, they bring a wide array of skills and contributions to the group. Through legitimate peripheral participation, new members acquire more leadership and educational skills and eventually play a more central role in the functioning of the committee. Similar formats have been suggested for junior faculty in clinician-educator roles [8, 9]. Few programs have extended these principles down to develop residents as medical educators.

Program evaluation

We conducted a program evaluation survey about our innovation. We received a waiver of exemption from the Hamilton Integrated Research Ethics Board. We surveyed 30 previous and current committee members who were involved over the last five years to determine if participation in the MAD planning committee was beneficial to their professional development as medical educators. The questions included in the survey are outlined in Table 2. The survey was designed and delivered electronically to their institutional email (kept open for alumni) via Google Forms (Mountainview, CA) with an explanatory email prompt. This email send-out was repeated one month later to increase the number of responses. All survey data was collected anonymously and stored on a password-protected Google Drive (Mountainview, CA).

**Table 2:** Program evaluation survey questions MAD - Multidisciplinary Academic Day; PGY - Postgraduate Year

Question	Question type
What is your level of training?	Select from: PGY1 to PGY5, fellow, staff, other
What was/is your residency program?	Short free-text answer
How many years did you participate on the MAD committee?	Select from 1-5, other
What goals did you have in joining the MAD committee?	Long free-text answer
Did you achieve your goal(s)?	Select from: yes/no/other
Please describe the experience you had as a member of the MAD committee.	Long free-text answer
What was your level of involvement in leadership roles prior to joining MAD?	Likert scale, 1 - no involvement ... to 7 - very involved with multiple roles
What was your level of involvement in medical education (MedEd) prior to joining MAD?	Likert 1 - no involvement ... to 7 - very involved with multiple roles
Was the MAD committee experience helpful for your career?	Likert scale 1 - not helpful at all ... to 7 - extremely helpful
How did your participation in the MAD committee impact your career?	Long free-text answer
Did participation in the committee increase your involvement in MedEd?	Long free-text answer
Was the MAD committee useful in developing your leadership skills?	Long free-text answer
What leadership skills, if any, did you develop as a result of your involvement with MAD?	Long free-text answer
Was involvement in the committee helpful in terms of networking with residents from other programs?	Likert scale 1 - not helpful at all ... to 7 - extremely helpful
How satisfied were you with your MAD experience?	Likert scale 1 - not satisfied at all ... to 7 - extremely satisfied
Would you recommend this experience to residents interested in leadership & MedEd?	Yes/no

Qualitative data was reviewed for overall themes by the author (AC - Alexander Chorley) and then coded using an integrated approach involving inductive and deductive frameworks [10]. The audit trail from this analysis including the subsequent codes was then reviewed by a second author (TC - Teresa Chan) to ensure accuracy. The analyzing author was the outgoing resident chair of the MAD planning committee and reviewing author was the current faculty advisor and also alumni of the committee. To ensure the accuracy of our results, we performed a member check by sending our analysis to the participants [11]. Responding participants indicated that the analysis of responses was consistent with their experiences and did not recommend any changes.

## Results

Responses were obtained from 19 of 30 (63.3%) committee members from 2012 to 2016. The demographic information of respondents is outlined in Table 3.

**Table 3:** Demographics of the participants at the time of program evaluation survey response

Category	Specifics	Number of participants (%)
Current post-graduate year	1	7 (36.8%)
	2	2 (10.5%)
	3	1 (5.3%)
	4	1 (5.3%)
	Fellow	4 (21.1%)
	Staff	4 (21.1%)
Discipline	Internal medicine and subspecialties	6 (31.6%)
	Emergency medicine	5 (26.3%)
	Pediatrics	3 (15.8%)
	Family medicine	3 (15.8%)
	Orthopedics	1 (5.3%)
	Anesthesia	1 (5.3%)
Years on the Multidisciplinary Academic Day committee	1	11 (57.9%)
	2	5 (26.3%)
	3	1 (5.3%)
	4	1 (5.3%)
	5	1 (5.3%)

The first set of questions addressed residents’ goals when joining the MAD planning committee. A summary is presented in Table 4. Several themes emerged, including the desire to gain medical education experience, learn about event planning and development, and improve resident education at McMaster University. Many others desired involvement with a multidisciplinary team. One respondent stated the goal as to “get more exposure to medical education in a multidisciplinary setting (outside my home program)”. The majority of respondents felt that involvement in MAD was useful for networking with residents from other programs, with 16 out of 19 (84.2%) ranking it as a five or greater on a seven-point Likert scale. Overall, 17 out of 19 (89.5%) felt that they had met their personal goals for joining the committee.

**Table 4:** Participants’ personal goals for joining the MAD planning committee MAD - Multidisciplinary Academic Day

Theme	% of respondents	n (total=19)
Expanding and developing their social network	47%	9
Gain experience with event planning	32%	6
Gain exposure/experience in medical education	21%	4
Improving residency education	16%	3
Develop leadership skills	16%	3
Serving the larger university and postgraduate training community	11%	2

Themes also emerged regarding residents’ experience while working with the committee. Many felt it was a positive experience, allowing them to acquire skills essential for medical education, ranging from event planning to curriculum development while working in a multidisciplinary team. One respondent stated that working on the committee included:

“...experience in brainstorming, curriculum development, committee-based planning and execution, working with speakers, developing evaluations and integrating feedback and suggestions into future events.”

When asked if participants were satisfied with their experience on MAD, 94.7% (18/19) respondents rated it as ≥5 on a seven-point Likert scale. The vast majority of respondents (94.7%) stated they would recommend this experience to other residents interested in leadership and medical education.

The majority of participants were significantly involved in leadership prior to joining the committee, with 84.2% (16/19) ranking their involvement as ≥4 on a seven-point Likert scale. Many found it useful in developing their leadership skills, with 68.4% (13/19) ranking it as ≥5 in terms of leadership development. In addition to identifying event planning and organization as major skills acquired, several other themes were identified. Leadership codes included leading a team, decision making, idea generation, delegation and public speaking. One respondent stated that it “*honed my ability to create a pitch for change, galvanize a group, create a systems-based change to an academic program, and build something for residents.*”

The respondents’ involvement in medical education prior to joining the committee was much more mixed. Nine out of 19 respondents (47.3%) reported a prior high (≥5, on a seven-point scale) involvement in medical education, whereas the remaining participants reported low levels of exposure. In our qualitative analysis of their feedback, we identified a number of themes about the MAD planning committee’s effect on their involvement in medical education. Themes identified by the participants included feelings of having a legitimate role in education, feeling empowered to contribute, having insight into medical education events, and using education as a foundation for an academic career. One respondent stated that *“...[n]ow doing a masters [sic] as my interest developed more.”* Another responded that involvement in the committee *“...contributed to [me] pursuing an academic career path.”* The majority of the MAD planning committee still felt that this experience increased their subsequent involvement in medical education, with 52.6% (10/19) respondents rating it as five or higher on a seven-point Likert scale.

The respondents were also asked about the impact of involvement in the MAD planning committee on their careers. Fifteen out of 19 (78.9%) felt it was helpful for their careers, citing the leadership skills acquired, insight into medical education, and appreciation for multidisciplinary relationships. One respondent stated:

“As an endocrinologist, I liaise with an interdisciplinary team, including dieticians, diabetes education nurses, lab techs, clinical research coordinators and medical office administrators. I feel that the MAD committee was helpful in terms of me getting my feet wet and liaising with individuals from a number of different disciplines.”

## Discussion

Our MAD planning committee is working at two levels to create an educational experience: 1) it is creating unique, resident-led educational content for institution-wide consumption; 2) it is exposing and training a cadre of residents to skills that are essential to being a leader and medical educator (e.g. needs assessments, program evaluation, curriculum design). In contrast to previous clinician-educator development and medical education fellowship programs, our program is designed to be intercalated with residency training, in an extra-curricular fashion for those who are interested in understanding what it takes to be an educator [8, 9]. The vast majority of the MAD planning committee’s alumni felt this was a beneficial experience and almost all participants felt they had met these goals during their time with the committee. Many joined in their first year of residency, hoping to gain exposure to medical education, then developed as medical educators, and subsequently became involved in a wider variety of academic roles.

For those wishing to design and implement similar educator-training programs, it may be worthwhile to consider both the intended and emergent conceptual frameworks that seem to best describe residents’ experiences in the MAD planning committee. We believe our committee was successful in developing medical educators because it operates according to key conceptual frameworks: Kolb’s model of experiential learning theory and the creation of a CoP [7, 12]. Residents begin with concrete experience (the first MAD day), after which they receive feedback in the form of program evaluation surveys and reflect on their observations. They then integrate these observations with the goals and objectives of the committee, conceptualizing ways to improve the next MAD day. Finally, they engage in active experimentation by implementing their new ideas in an attempt to improve their educational product by soliciting and incorporating program evaluation into their processes [13].

Additionally, the CoP aspect of the committee is also essential for resident growth and development as educators. This CoP allows residents to essentially apprentice in curriculum development, learning from each other as well as an experienced faculty advisor, acquiring skills that will benefit them in their future academic careers. It also allows networking to occur between residents from different specialties, creating opportunities for future collaboration.

Limitations

Many of the committee members had only one year of experience on the committee, and we felt it was too early to judge its impact on their careers. By using their institutional email address to contact former members, several may have been missed if they no longer utilize that account. The use of a single author to analyze and code the data may have been a limitation; however, some experts argue that this is sufficient and preferable when a relationship with the participants is an important factor in the analysis, and we also utilized other methods to ensure rigor and trustworthiness of our analysis (e.g. audit trail, member check) [10].

## Conclusions

Involvement in the MAD planning committee is a useful way for residents to acquire skills, develop their interest in medical education and create multidisciplinary relationships. Residents involved in this committee have gone on to pursue master’s level training in education as well as assume leadership positions in education at academic institutions. Next steps within this line of work could be to determine whether the program leads to increased leadership and interest in education, or if it develops and facilitates those who have already been self-identified.
